# Intracranial Efficacy of Crizotinib and Postprogression Therapeutic Strategies in Advanced c‐ros Oncogene 1 (ROS1)‐Positive Non–Small Cell Lung Cancer (NSCLC), a Multicenter Real‐World Study

**DOI:** 10.1002/mco2.70854

**Published:** 2026-07-13

**Authors:** Zihua Zou, Xiaobin Zheng, Panwen Tian, Zhe Liu, Jie Hu, Yong Fang, Yang Xia, Feng Ye, Tangfeng Lv, Li Li, Diansheng Zhong, Jin Zhou, Qian Chu, Meiqi Shi, Chengbo Han, Baoshan Cao, Dingzhi Huang, Hui Guo, Gen Lin

**Affiliations:** ^1^ Department of Thoracic Oncology Clinical Oncology School of Fujian Medical University Fujian Cancer Hospital Fujian China; ^2^ Department of Medical Oncology Beijing Tuberculosis and Thoracic Tumor Research Institute Beijing Chest Hospital Capital Medical University Beijing China; ^3^ Department of Pulmonary and Critical Care Medicine Lung Cancer Center Precision Medicine Key Laboratory of Sichuan Province West China Hospital Sichuan University Chengdu China; ^4^ Shanghai Geriatric Center Zhongshan Hospital Fudan University Shanghai China; ^5^ Department of Medical Oncology Sir Run Run Shaw Hospital Zhenjiang University School of Medicine Hangzhou China; ^6^ Department of Respiratory and Critical Care Medicine Second Affiliated Hospital of Zhejiang University School of Medicine Hangzhou China; ^7^ Department of Medical Oncology Xiamen Key Laboratory of Antitumor Drug Transformation Research School of Medicine the First Affiliated Hospital of Xiamen University Xiamen China; ^8^ Department of Respiratory Medicine Affiliated Jinling Hospital Medical School of Nanjing University Nanjing China; ^9^ Department of Respiratory Disease Daping Hospital Army Medical University Chongqing China; ^10^ Department of Medical Oncology Tianjin Medical University General Hospital Tianjin China; ^11^ Department of Medical Oncology Sichuan Clinical Research Center for Cancer Sichuan Cancer Center Sichuan Cancer Hospital & Institute Affiliated Cancer Hospital of University of Electronic Science and Technology of China Chengdu China; ^12^ Department of Oncology Tongji Hospital Tongji Medical College Huazhong University of Science and Technology Wuhan China; ^13^ Department of Medical Oncology Jiangsu Cancer Hospital and Jiangsu Institute of Cancer Research and Affiliated Cancer Hospital of Nanjing Medical University Nanjing China; ^14^ Department of Oncology Shengjing Hospital of China Medical University Shenyang China; ^15^ Cancer Center Department of Medical Oncology and Radiation Sickness Peking University Third Hospital Beijing China; ^16^ Department of Thoracic Oncology Tianjin Medical University Cancer Institute & Hospital Tianjin China; ^17^ Department of Medical Oncology the Second Affiliated Hospital of Xi'an Jiaotong University Xi'an China

**Keywords:** c‐ros oncogene 1, central nervous system efficacy, Crizotinib, non–small cell lung cancer, subsequent treatments

## Abstract

Intracranial efficacy of crizotinib and optimal postprogression therapies in c‐ros oncogene 1 (ROS1)‐positive non–small cell lung cancer(NSCLC) remain under‐explored. We performed a multicenter real‐world study across 17 Chinese hospitals (2015–2024) in 325 ROS1‐positive NSCLC patients to evaluate the intracranial efficacy of crizotinib and postprogression treatment strategies. Crizotinib achieved an overall response rate(ORR) of 78.5% and a median progression‐free survival (PFS) of 22.4 months. Patients with baseline brain metastases had a 66.7% intracranial response rate and a median central nervous system (CNS)‐PFS of 22.2 months, demonstrating intracranial benefit. After progression, next‐generation ROS1 inhibitors (active against the G2032R resistance mutation, for example, taletrectinib and repotrectinib) significantly prolonged PFS compared to older‐generation targeted agents (median 13.9 vs. 4.4 months), especially among patients with secondary ROS1 resistance mutations (ORR 66.7% vs. 14.3%). However, due to greater availability in China, earlier agents like lorlatinib and entrectinib were commonly used after crizotinib failure. In our cohort, lorlatinib demonstrated superior efficacy to entrectinib, with a trend toward longer PFS and higher overall (34% vs. 7%) and intracranial (50% vs. 0%) response rates. This large real‐world study confirms crizotinib's intracranial benefit and underscores the importance of next‐generation ROS1 inhibitors and older‐generation agents like lorlatinib—for improved patient outcomes after crizotinib resistance.

## Graphical abstract

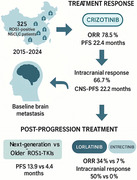

## 1 Introduction

The c‐ros oncogene 1 (ROS1) encodes a receptor tyrosine kinase [1], whose rearrangement with various fusion partners leads to dysregulated downstream signaling, serving as a potent oncogenic driver mutation. ROS1 rearrangements are detected in approximately 1%–2% of non–small cell lung cancer (NSCLC) cases, with comparable prevalence in both Asian and Western populations. Patients harboring ROS1 fusions often exhibit similar clinicopathologic characteristics, including a younger age at diagnosis, adenocarcinoma histology, and a history of no or light smoking [2]. Clinically, ROS1‐positive NSCLC is associated with aggressive disease behavior, high metastatic potential, and a marked propensity for central nervous system (CNS) involvement, all of which significantly affect patient prognosis.

Several targeted therapies have demonstrated significant efficacy in the treatment of ROS1‐positive NSCLC. For instance, crizotinib [3, 4, 5, 6, 7, 8], entrectinib [9, 10, 11], and lorlatinib [12, 13, 14] have shown response rates of approximately 65%–80% and progression‐free survival (PFS) of 16–24 months. Next‐generation ROS1 inhibitors, such as repotrectinib [15, 16] and taletrectinib [17], which are effective against the G2032R resistance mutation, exhibit superior efficacy, with first‐line PFS exceeding 36 months and high response rates in patients harboring the G2032R mutation following disease progression with crizotinib. However, owing to limitations in accessibility and cost, crizotinib and entrectinib remain the most widely used ROS1 inhibitors in real‐world settings in the Chinese population.

Crizotinib, a multitarget (anaplastic lymphoma kinase [ALK]/ROS1//(mesenchymal–epithelial transition factor [MET]) small‐molecule tyrosine kinase inhibitor (TKI), widely recognized as one of the most successful therapeutic agents for NSCLC has been extensively evaluated in numerous clinical trials [3, 4, 5, 6, 7, 8], retrospective studies [18, 19, 20, 21] and systematic literature reviews [22]. Translational studies have further investigated various biomarkers associated with crizotinib efficacy [23, 24, 25, 26, 27], including different fusion partners, programmed death‐ligand 1(PD‐L1) expression levels, and co‐mutation status. Several researchers have described the mechanisms underlying crizotinib resistance have also been described by several researchers [28, 29, 30]. However, certain issues remain incompletely addressed and require further investigation.

First, clinical trials and real‐world studies have provided limited detailed data regarding the intracranial efficacy of crizotinib in patients with ROS1‐positive NSCLC. Because ALK‐ and ROS1‐positive NSCLC share clinicopathological features, highly homologous tyrosine kinase domains, and sensitivity to crizotinib, these two groups are often compared. Prior studies have reported inadequate intracranial responses and limited CNS protective effects [31, 32, 33, 34]. This may not directly translate to ROS1‐positive disease, since ROS1‐positive tumors appear less prone to CNS involvement [28] and more sensitive to crizotinib (median PFS generally <1 year in advanced ALK‐positive NSCLC vs. 15–22 months in ROS1‐rearranged NSCLC). Collectively, these results suggest that crizotinib may achieve better intracranial control in ROS1‐positive NSCLC than in ALK‐positive NSCLC; however, ROS1‐specific intracranial data remain sparse, so this hypothesis is still speculative.

Second, because resistance is almost inevitable, postcrizotinib treatment selection is a key clinical issue. Although next‐generation ROS1 inhibitors [16, 17] have shown favorable activity in later lines, their use is limited by cost and access. Therefore, more accessible non‐next‐generation options, such as entrectinib and lorlatinib, are often used after crizotinib resistance in real‐world settings. Some studies suggest their moderate efficacy [11, 12, 13], however, head‐to‐head comparative data between these two drugs in this setting are lacking. In addition, immunotherapy (IO) monotherapy has shown limited benefits in NSCLC with actionable alterations (e.g., epidermal growth factor receptor (EGFR), ALK, ROS1, rearranged during transfection) [35, 36]. Although IO‐based combinations may be effective after EGFR‐TKI resistance [37, 38], it remains unclear whether they outperform the standard chemotherapy for ROS1‐positive NSCLC.

Given these unresolved questions, we conducted a multicenter retrospective analysis aimed at evaluating the intracranial efficacy of crizotinib in patients with ROS1‐positive NSCLC and to identify optimal therapeutic strategies following crizotinib progression.

## 2 Results

### 2.1 Baseline Characteristics in Overall Population

In total, 325 patients initially treated with crizotinib were enrolled in this study. The detailed baseline characteristics are summarized in Table 1. The majority of the enrolled patients were female, had no smoking history, and presented with a good performance status. Molecular diagnostic methods, predominantly reverse transcription‐polymerase chain reaction (RT‐PCR, *n* = 140) and next‐generation sequencing (NGS, *n* = 122), were used to confirm ROS1 fusion status. Fusion partner data were available for 145 patients either at baseline or at the time of progression, with CD74 (*n* = 78), EZR (*n* = 24), and SDC4 (*n* = 23) identified as the most common fusion partners. PD‐L1 expression data at baseline were reported in 121 patients, with PD‐L1‐negative cases (39.6%) being slightly more common than those with PD‐L1 expression levels of 1%–49% (30.6%) or ≥50% (29.8%). Regarding disease burden, 276 patients (84.9%) had stage IV disease, 159 (48.9%) had extrathoracic metastases, and 51 (15.7%) had distant metastases involving three or more organs. Metastases involving the CNS, liver, and bone were observed in 24.0%, 8.6%, and 25.8% of the patients, respectively.

**TABLE 1:** Baseline characteristics.

	*N* = 325
Age	Median 58 (Range 15–89)
Sex	
Male	136 (41.8%)
Female	189 (57.2%)
Smoking history	
Yes	62 (19.1%)
No	263 (80.9%)
Pathology	
Adenocarcinoma	316 (97.3%)
	
Squamous cell carcinoma	5 (1.5%)
other	4 (1.2%)
ECOG PS	
0–1	306 (94.2%)
≥2	19 (5.8%)
Stage	
IIIB or IIIC	49 (15.1%)
IV	276 (84.9%)
Diagnostic methods	
RT‐PCR	140 (42.1%)
ARMS	34 (10.5%)
NGS	122 (37.5%)
FISH	29 (8.9%)
ROS1 fusion partner	
CD74	78 (24%)
EZR	24 (7.4%)
SDC4	23 (7.1%)
SLC34A2	10 (3.1%)
Other	10 (3.1%)
Unknown	180 (55.3%)
PD‐L1 expression level	
Negative	48 (14.8%)
1–49%	37 (11.2%)
≥50%	36 (11.2%)
Unknown	204 (62.8%)
Extrathoracic metastases	
Yes	159 (48.9%)
No	166 (51.1%)
Brain metastases	
Yes	78 (24%)
No	247 (76%)
Liver metastases	
Yes	28 (8.6%)
No	297 (91.4%)
Bone metastases	
Yes	84 (25.8%)
No	241 (74.2%)
Distant organs involved	
≤2	274 (84.3%)
≥3	51 (15.7%)
Target lesion	
Yes	312 (96.0%)
No	13 (4.0%)
Prior chemotherapy	
Yes	56 (17.2%)
No	269 (82.8%)

### 2.2 Efficacy of Crizotinib and Potential Clinical or Molecular Factors That Impact the Efficacy of Crizotinib

**FIGURE 1:**
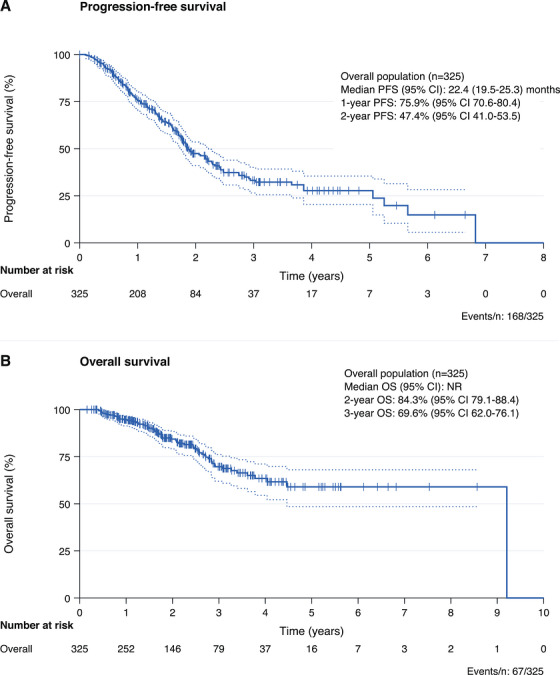
Progression‐free survival (PFS) and Overall survival (OS) Kaplan–Meier (K–M) curves in the overall population upon the treatment of crizotinib. (A) PFS in the overall population, CI, confidence interval. (B) OS in the overall population. NR, not reached.

Among patients with target lesions (*n* = 312), 78.5% (95% confidence intervals [CI]: 73.5%–83.0%) achieved an objective response to crizotinib. With a median follow‐up of 22.5 months (range, 1.9–119 months), 168 patients experienced disease progression or death, of whom 57.7% had isolated extracranial progression, 26.8% experienced isolated intracranial progression, and 14.5% had both intracranial and extracranial progression. The median PFS was 22.4 months (95% CI: 19.5–25.3 months), and the 1‐year, 2‐year, and 3‐year PFS rates were 75.5% (95% CI: 70.2%–80.1%), 46.8% (95% CI: 40.4%–53.0%), and 33.2% (95% CI: 26.5%–40.0%) (Figure 1A), respectively. The median overall survival (OS) was not reached using this data cutoff value. The OS rates at 1, 2, and 3 years were 94.2% (95% CI: 90.9%–96.4%), 83.8% (95% CI: 78.4%–87.9%), and 69.6% (95% CI: 62.0%–76.1%) (Figure 1B), respectively.

Univariate analysis of the clinical factors potentially affecting crizotinib efficacy showed that sex, smoking history, age, performance status, prior chemotherapy lines, and presence of brain or bone metastases were not significantly associated with PFS. However, stage IV disease, liver metastases, extrathoracic metastases, and involvement of ≥3 distant organs were identified as negative prognostic factors.

**FIGURE 2:**
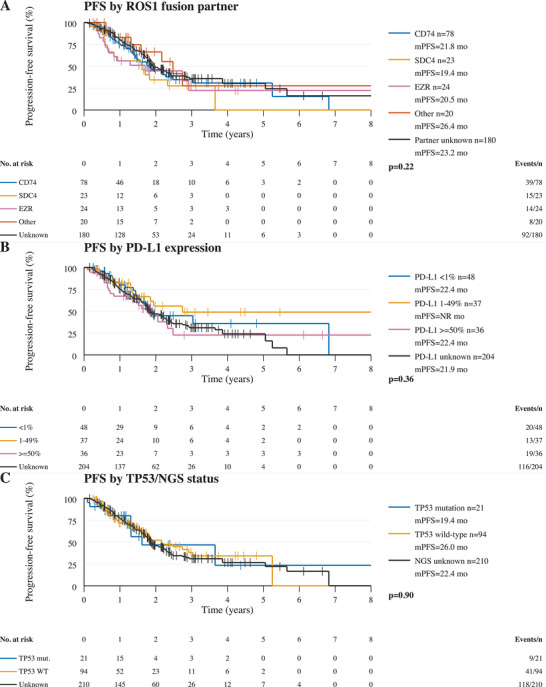
PFS K–M curves in patients with different molecular characteristics upon the treatment of crizotinib. (A) PFS in patients with different ROS1 fusion partners. (B) PFS in patients with different PD‐L1 expression levels. (C) PFS in patients with different TP53 mutation status.

We further evaluated potential predictive biomarkers by focusing on three parameters frequently assessed before targeted therapy: PD‐L1 expression, fusion partner, and tumor protein p53 (TP53) mutation status. Owing to the variability in the NGS panels used (data extracted from electronic medical record systems [EMRs]), we selected TP53 as the most frequently co‐mutated gene for further analysis. Patients lacking detailed information regarding these biomarkers were categorized into the “unknown” subgroup. In univariate analyses, no significant differences in PFS were observed among patients with different fusion partners (CD74 vs. SDC4 vs. EZR vs. others vs. unknown: 21.8 m vs. 19.4 m vs. 20.5 m vs. 29.6 m vs. 23.2 m; *p* = 0.22, Figure 2A). Similarly, PD‐L1 expression levels showed no significant impact on crizotinib efficacy (negative vs. 1%–49% vs. ≥50% vs. unknown: 22.4 m vs. NR vs. 22.4 m vs. 21.9 m; *p* = 0.36, Figure 2B). Additionally, TP53 mutation status did not significantly affect PFS (TP53 mutated vs. wild‐type vs. unknown: 19.4 m vs. 26.0 m vs. 22.4 m; *p* = 0.90, Figure 2C).

Covariates with *p*‐values <0.1 in univariate analyses were included in the multivariate model. Multivariate analysis demonstrated that the disease stage was the only independent factor significantly associated with crizotinib efficacy, indicating that patients with stage IV disease had less favorable clinical outcomes. The results of the univariate and multivariate analyses are summarized in Table 2.

**TABLE 2:** Univariate and multivariate analysis of PFS during the treatment of crizotinib covariates with *p* < 0.1 in the univariate analysis were included in the multivariate model.

variables	Univariate analysis	Multivariate analysis
Sex	HR = 0.91 (95% CI: 0.66–1.24)	
Male vs. female	*p* = 0.538	
Age	HR = 0.91(95% CI: 0.81–1.11)	
<65 vs. ≥65	*p* = 0.492	
Smoking history	HR = 1.02 (95% CI: 0.84–1.25)	
No vs. Yes	*p* = 0.812	
ECOG PS	HR = 0.77 (95% CI: 0.58–1.04)	HR = 0.68 (95% CI: 0.37–1.25)
0–1 vs. ≥2	*p* = 0.083	*p* = 0.212
Stage	HR = 0.66 (95% CI: 0.51–0.86)	HR = 0.50 (95% CI: 0.28–0.87)
III vs. IV	*p* = 0.0017	*p* = 0.015
Prior chemotherapy	HR = 1.0 (95% CI: 0.83–1.22)	
No vs. Yes	*p* = 0.95	
Brain metastases	HR = 0.86 (95% CI: 0.73–1.02)	HR = 1.24 (95% CI: 0.77–2.00)
No vs. Yes	*p* = 0.08	*p* = 0.372
Liver metastases	HR = 0.73 (95% CI: 0.57–0.94)	HR = 0.84 (95% CI: 0.81–1.11)
No vs. Yes	*p* = 0.01	*p* = 0.602
Bone metastases	HR = 0.89 (95% CI: 0.75–1.06)	
No vs. Yes	*p* = 0.18	
Extrathoracic metastases	HR = 0.82 (95% CI: 0.70–0.95)	HR = 0.87 (95% CI: 0.58–1.30)
No vs. Yes	*p* = 0.009	p = 0.504
Distant organs involved	HR = 0.75 (95% CI: 0.62–0.91)	HR = 0.66 (95% CI: 0.37–1.17)
≤2 vs. ≥3	*p* = 0.0027	p = 0.156
PD‐L1 level		
<1% vs. 1%–49% vs. ≥50 vs. unknown	*p* = 0.36	
Fusion partner		
CD74 vs. SDC4 vs. EZR vs. other vs. unknown	*p* = 0.22	
TP53 status		
Mutated vs. wild‐type vs. unknown	*p* = 0.9	

### 2.3 Intracranial Efficacy of Crizotinib

At baseline, 78 patients presented with CNS metastases before the initiation of crizotinib treatment. Among them, 19 patients received radiotherapy (RT) within 3 months before or during crizotinib administration, and 20 patients had more than five brain metastases. Measurable intracranial lesions were identified in 54 patients, with the median sum of the longest diameters being 22 mm (range, 10–55 mm).

**FIGURE 3:**
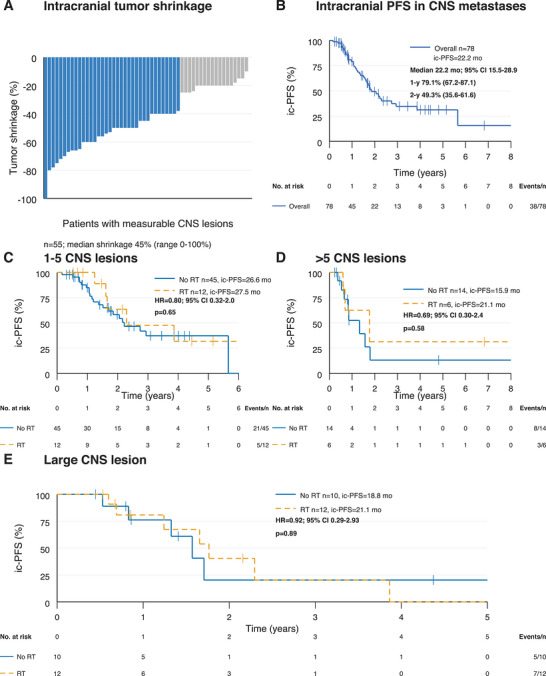
Intracranial efficacy of crizotinib. (A) Waterfall plot for CNS efficacy of crizotinib in patients with measurable intracranial metastases. (B) Intracranial Progression‐free survival (ic‐PFS) of crizotinib in patients with baseline CNS metastases. (C) IC‐PFS in patients with 1–5 CNS metastases with or without local therapy. (D) IC‐PFS in patients with >5 CNS metastases with or without local therapy. (E) IC‐PFS in patients with large CNS metastases with or without local therapy.

Among patients with measurable CNS lesions, the intracranial objective response rate (CNS‐ORR) was 66.7% (95% CI: 49.6%–77.2%), and the median tumor shrinkage rate was 45% (range 0%–100%) (Figure 3A). At the time of data cutoff, progression was documented in 51 patients, with intracranial progression being the predominant site (74.5%), whereas CNS progression was only reported in 28.8% of the patients without baseline intracranial metastases. Median CNS‐PFS and median duration of intracranial response were 22.2 months (95% CI: 15.5–28.9 months) (Figure 3B) and 24.7 months (95% CI: 16.8–32.7 months) for patients with baseline CNS metastases, respectively. Figure S2A–C illustrates representative examples of patients who experienced a significant reduction in intracranial lesions during crizotinib treatment (none of the three patients had received brain RT).

We further analyzed the effect of simultaneous brain RT. Although RT did not significantly improve the CNS‐ORR compared to crizotinib alone (71.4% [10/14] vs. 62.5% [25/40], *p* = 0.339), it significantly increased the proportion of patients achieving greater than 50% intracranial tumor shrinkage (71.4% vs. 37.5%, *p* = 0.035). However, simultaneous RT did not confer additional survival benefits (CNS‐PFS: 22.2 months with RT vs. 22.4 months without RT; hazards ratio [HR] = 0.92, 95% CI: 0.49–1.70, *p* = 0.80).

Given the clinical practice of preferentially using RT in patients with higher intracranial tumor burden, subgroup analyses were performed. Our results indicated that RT did not significantly prolong CNS‐PFS, regardless of whether patients had oligometastatic (1–5 lesions) or extensive (>5 lesions) CNS disease (1–5 lesions: 27.5 vs. 26.6 months, HR = 0.92, 95% CI: 0.44–1.95, *p* = 0.82, Figure 3C; >5 lesions: 21.1 vs. 15.8 months, HR = 0.78, 95% CI: 0.41–1.92, *p* = 0.58, Figure 3D). Additionally, no significant CNS‐PFS advantage was observed with RT in patients harboring large CNS metastases (sum of the longest intracranial lesion diameters ≥20 mm: 21.1 vs. 18.8 months, HR = 0.92, 95% CI: 0.29–2.91, *p* = 0.88, Figure 3E). These findings indicate that the addition of RT may not substantially improve the survival outcomes in patients with a significant CNS tumor burden receiving crizotinib.

### 2.4 Potential Resistant Mechanism and Subsequent Therapy Following the Progression of Crizotinib

**FIGURE 4:**
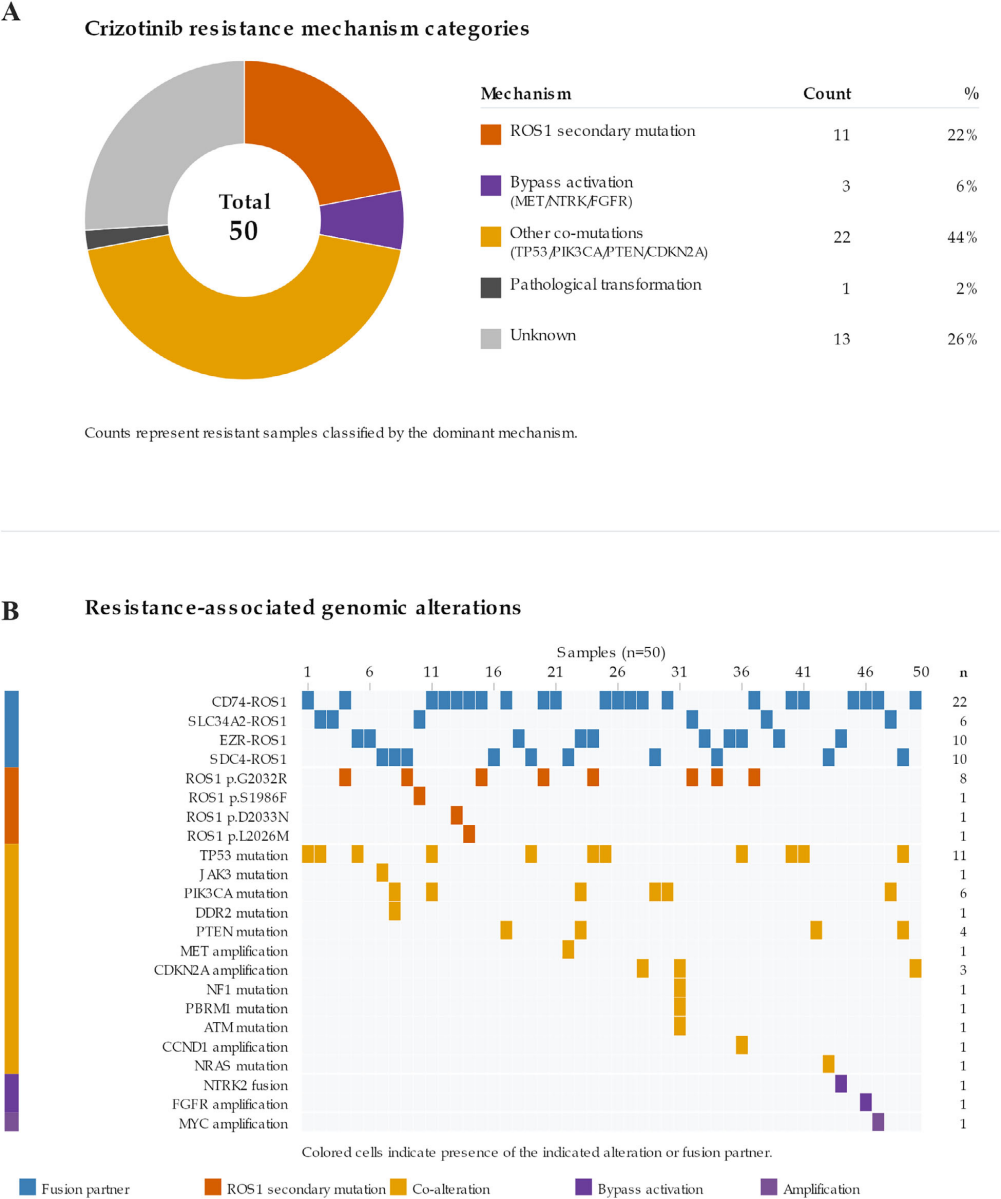
Molecular characteristics of the resistance to crizotinib. (A) Possible resistant mechanism categories of crizotinib. (B) Detailed NGS results for patients with rebiopsy at the resistance of crizotinib.

A total of 168 patients experienced disease progression or death after crizotinib treatment. Among these patients, detailed re‐biopsy and NGS results obtained at the time of progression were available for 50 (tissue biopsy, *n* = 30; liquid biopsy, including plasma or pleural effusion, *n* = 20). Potential resistance mechanisms are summarized in Figure 4A,B. Secondary mutations in the ROS1 kinase domain were identified in 22% (11/50) of patients, including G2032R (*n* = 8), S1986F, L2026M, and D2033N (each n = 1). Additionally, bypass pathway activation was detected in three patients, including MET amplification, neurotrophic tropomyosin receptor kinase (NTRK) fusion, and fibroblast growth factor receptor (FGFR) amplification. Pathological transformation to squamous cell carcinoma occurred in one patient. Common genomic alterations at progression also included TP53 mutations, phosphatase and tensin homolog (PTEN) mutations, phosphatidylinositol‐4,5‐bisphosphate 3‐kinase catalytic subunit alpha (PIK3CA) activation, and cyclin‐dependent kinase inhibitor 2A (CDKN2A) amplification. The frequency of secondary ROS1 mutations observed in this study was lower than previously reported, possibly because of several factors. First, patients with isolated CNS progression underwent extracranial rather than intracranial lesion biopsy. Second, a relatively high proportion (25%) of liquid biopsies were used in our study, which generally have lower sensitivity compared to tissue‐based analyses.

Detailed medical records of subsequent therapies following crizotinib progression are available for 140 patients (Table 3). Physicians typically favored local therapy combined with continued crizotinib treatment for patients with localized or oligometastatic disease progression. For patients with systemic progression, subsequent therapy with alternative ROS1‐TKIs was the most common strategy. In contrast, chemotherapy‐ or immunotherapy‐based combination regimens were generally reserved for patients whose disease had progressed after multiple lines of targeted therapies. Twenty‐one patients received local therapy combined with continued crizotinib after local progression (intracranial progression, *n* = 17; extracranial progression, *n* = 4). The median duration of disease control was 7.4 months (95% CI: 2.3–12.6 months).

**TABLE 3:** Subsequent therapy following the progression of crizotinib.

	Number of patients
First‐line treatment after crizotinib	*N* = 140
Local therapy plus crizotinib	21
Next‐generation ROS1‐TKI	8
Non next‐generation ROS1‐TKI	71
Chemo+anti‐VEGF	20
Chemo alone	12
IO‐based combination	6
IO alone	1
ROS1+MET TKI	1
Second‐line treatment after crizotinib	*N* = 57
Next‐generation ROS1‐TKI	4
Non next‐generation ROS1‐TKI	22
Chemo+anti‐VEGF	18
Chemo alone	9
IO‐based combination	4
Third‐line treatment after crizotinib	*N* = 22
Next‐generation ROS1‐TKI	1
Non next‐generation ROS1‐TKI	4
Chemo+anti‐VEGF	6
Chemo alone	2
IO‐based combination	8
IO alone	1
Fourth‐line treatment after crizotinib	*N* = 2
Non‐next‐generation ROS1‐TKI	2

**FIGURE 5:**
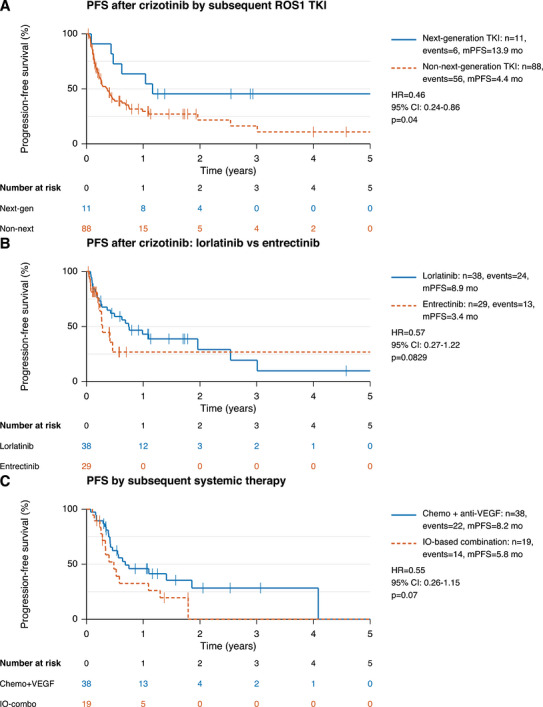
PFS K–M curves among different subsequent therapies after progression of crizotinib. (A) PFS for next‐generation ROS1 TKIs vs non‐next‐generation ROS1 TKIs after progression of crizotinib. (B) PFS for lorlatinib vs. entrectinib after progression of crizotinib. (C) PFS for chemo+anti‐VEGF vs IO‐based combination treatment in later‐line setting.

Therefore, we evaluated the efficacy of alternative ROS1 inhibitors following resistance to crizotinib. A total of 99 patients received ROS1‐TKIs as their first targeted therapy following the progression of crizotinib treatment. Patients who initially received chemotherapy after crizotinib progression and subsequently underwent treatment with another ROS1‐TKI were also included in this group. Next‐generation ROS1‐TKIs (repotrectinib, taletrectinib, TL‐139; *n* = 11) exhibited superior efficacy compared with non‐next‐generation ROS1‐TKIs (*n* = 88), demonstrating significantly longer PFS (PFS: 13.9 vs. 4.4 months, HR = 0.46, 95% CI: 0.24–0.86, *p* = 0.04; Figure 5A) and higher objective response rates among patients with measurable lesions (70% vs. 20%, *p* = 0.002). Notably, all three patients with measurable CNS lesions responded to next‐generation ROS1‐TKIs, highlighting their robust intracranial activity. In contrast, non‐next‐generation TKIs showed only moderate intracranial efficacy (CNS ORR = 34.3%, 12/35) in patients with CNS target lesions. Moreover, among patients harboring the G2032R mutation, next‐generation ROS1‐TKIs demonstrated promising efficacy, with an objective response rate (ORR) of 66.7% (2/3), suggesting a high sensitivity to solvent‐front mutations. Conversely, non‐next‐generation TKIs exhibited limited activity against secondary ROS1 resistance mutations (G2032R, *n* = 4; D2033N, *n* = 1; L2026M, *n* = 1; and S1986F, *n* = 1). Among these patients, only one harboring the L2026M mutation responded to lorlatinib, whereas the others showed progressive disease. These results indicate that next‐generation ROS1‐TKIs should be strongly recommended for patients with secondary ROS1 mutations following crizotinib resistance.

Given cost and accessibility considerations, entrectinib and lorlatinib are the most commonly used ROS1 inhibitors in China following crizotinib resistance. Therefore, we evaluated the efficacy of these two drugs in this context. Compared with entrectinib, lorlatinib demonstrated numerically longer PFS (8.9 vs. 3.4 months, HR = 0.57, 95% CI: 0.27–1.22, *p* = 0.0829, Figure 5B) and significantly higher ORR (34.2% [13/38] vs. 6.9% [2/29], *p* = 0.002). Furthermore, lorlatinib exhibited notably superior intracranial efficacy compared with entrectinib (CNS‐ORR in patients with measurable CNS lesions and no simultaneous radiotherapy: 50% [8/16] vs. 0% [0/6], *p* = 0.023). These findings suggest that lorlatinib is preferred over entrectinib as a subsequent treatment option, particularly in patients with CNS metastases.

Currently, IO‐based combination treatments are increasingly applied in real‐world clinical practice for patients harboring driver oncogene mutations. Therefore, we investigated the efficacy of IO‐based combination therapy in patients with ROS1‐positive NSCLC undergoing later‐line treatment. However, this strategy (*n* = 17) demonstrated no additional benefit compared to traditional chemotherapy plus antivascular endothelial growth factor (VEGF) therapy (*n* = 38), showing similar ORR (63.2% vs. 50%, *p* = 0.41) and PFS (5.8 months vs. 8.2 months; HR = 1.82, 95% CI: 0.86–3.83, *p* = 0.07; Figure 5C). These findings suggest that IO‐based combination therapies have limited clinical value in this population.

## 3 Discussion

Targeted therapies for ROS1‐positive NSCLC are evolving rapidly, with both first‐ (e.g., crizotinib [3, 4, 5, 6, 7, 8], entrectinib [9, 10, 11], and so on) and next‐generation (e.g., repotrectinib [15, 16] and taletrectinib [17]) TKIs demonstrating notable clinical efficacy. Despite the establishment of multiple drugs as standard treatments in this field, crizotinib and entrectinib remain the most commonly used agents in mainland China, owing to their availability and affordability. Although crizotinib has been extensively investigated in various clinical trials and translational studies, we conducted this real‐world study to evaluate its intracranial efficacy and analyze treatment outcomes following crizotinib resistance, aiming to provide additional insights in this clinical setting. To the best of our knowledge, this study represents the largest multicenter analysis to date, providing the first detailed report on the intracranial efficacy of crizotinib and comprehensively describing the effectiveness of subsequent therapeutic strategies following crizotinib progression.

In this study, we evaluated the overall efficacy of crizotinib in a real‐world setting and investigated the clinical characteristics and molecular biomarkers potentially associated with treatment response. Consistent with previous findings [3, 4, 5, 6, 7, 8, 18, 19, 20, 21, 22, 27], crizotinib demonstrated favorable clinical efficacy in patients with advanced ROS1‐positive NSCLC. Notably, the median PFS was somewhat longer than that reported in previous studies, possibly because of the lower proportion of patients with extensive extrathoracic metastases enrolled in our cohort. Univariate analysis revealed that a higher tumor burden, including stage IV disease, the presence of extrathoracic metastases, liver metastases, and metastases involving three or more distant organs, was associated with a poorer prognosis, whereas multivariate analysis only showed that disease stage was correlated with the efficacy of crizotinib, which was in line with our expectations. Consistent with previous reports [23], PD‐L1 expression levels were not associated with crizotinib efficacy, further suggesting that PD‐L1 expression may not serve as an effective biomarker for predicting the crizotinib response. However, in contrast to previous studies, we did not observe significant differences in crizotinib efficacy among different ROS1 fusion partners [24, 25, 26]; this discrepancy may be attributable to our limited sample size. Moreover, our analysis revealed no significant association between TP53 co‐mutations and crizotinib efficacy, in contrast to previous reports [25]. This discrepancy may be attributed to the limited sample size and variability in the testing platforms, leading to inconsistent detection sensitivity in our study. Another potential reason is that only specific subsets of TP53 mutations negatively impact the efficacy of targeted therapy; however, our study did not include a detailed characterization of the TP53 mutation subtypes.

Given that ALK‐positive and ROS1‐positive NSCLC share similar clinicopathological characteristics, the tyrosine kinase domains of ALK and ROS1 are highly homologous, and crizotinib is active against both targets, these two patient populations are frequently compared in parallel. However, previous evidence indicating poor intracranial efficacy of crizotinib in ALK‐positive NSCLC [31, 32, 33, 34] may not necessarily extend to ROS1‐positive NSCLC, as existing studies suggest that ROS1‐positive NSCLC exhibits comparatively lower CNS aggressiveness [28]. Moreover, in terms of overall efficacy, crizotinib appears to be more effective in ROS1‐rearranged NSCLC than in ALK‐rearranged disease (the median PFS with crizotinib in advanced ALK‐positive NSCLC is generally <1 year, whereas in ROS1‐positive NSCLC it can reach 15–22 months). Taken together, these observations suggest that the intracranial efficacy of crizotinib in ROS1‐positive NSCLC may also be more favorable than that observed in ALK‐positive NSCLC. However, as detailed data specifically characterizing the intracranial activity of crizotinib in ROS1‐positive NSCLC are currently lacking, this hypothesis remains speculative. Therefore, we conducted a detailed evaluation of intracranial response to crizotinib in our ROS1‐positive cohort. Interestingly, we observed better intracranial efficacy than previously anticipated, with the CNS‐ORR exceeding 60% and CNS‐PFS surpassing 20 months among patients presenting with baseline CNS metastases. However, it remains uncertain whether this difference reflects pharmacological factors, such as greater sensitivity of the ROS1 target to crizotinib, or tumor‐biological factors, such as lower intrinsic aggressiveness of ROS1‐positive NSCLC. It should be noted that we chose the response evaluation criteria in solid tumors (RECIST) criteria rather than modified RECIST (mRECIST) or response assessment in neuro‐oncology brain metastases (RANO‐BM) to assess intracranial efficacy because of their broader applicability and ease of use in clinical practice, despite the potential for underestimating the intracranial tumor burden. Moreover, the CNS evaluation intervals were not standardized for all patients, which might have led to some bias. Given that RT is often the preferred approach for patients with a significant intracranial disease burden in real‐world clinical practice, we specifically analyzed the CNS outcomes in the relevant subgroups. Our results showed that simultaneous RT did not meaningfully prolong CNS‐PFS in patients with multiple CNS lesions or large intracranial metastases. These findings should be interpreted cautiously, given the limited sample size. Additionally, we did not differentiate between the RT methods (whole‐brain radiotherapy [WBRT] and stereotactic body radiotherapy [SBRT]), which could introduce confounding effects. Given that SBRT has become the predominant approach for brain metastases, including those in patients with 5–10 intracranial lesions, it is reasonable to infer that SBRT was more frequently employed than WBRT in our cohort. It should be particularly emphasized that some patients would experience out‐of‐field intracranial progression after SBRT. Thus, although SBRT may improve local control at irradiated sites, out‐of‐field progression could partially account for the absence of a significant CNS‐PFS benefit in the local therapy group in this study. We also found that CNS progression events occurred significantly more often in patients with baseline CNS metastases than in those without (74.5% vs. 28.8%, respectively). However, we did not systematically characterize intracranial failure patterns, which represents an important direction for future research. As noted above, out‐of‐field progression is common after SBRT, which also reflects the limited whole‐brain protection conferred by targeted agents alone. Taken together, these observations underscore the clinical need to prioritize TKIs with improved blood‐brain barrier penetration and superior intracranial activity when treating this vulnerable subpopulation.

Furthermore, we performed an in‐depth analysis of the efficacy of subsequent therapies after crizotinib treatment. We directly compared the efficacy of older and next‐generation ROS1 inhibitors, which has rarely been reported before. As anticipated, next‐generation ROS1 inhibitors exhibited markedly superior efficacy compared with non‐next‐generation TKIs in our study. In patients with secondary ROS1 mutations, treatment with first‐generation TKIs yielded poor outcomes, whereas next‐generation inhibitors demonstrated promising efficacy, particularly against the G2032R mutation, which frequently occurs after crizotinib resistance. These results align with previous findings [12, 15, 16, 17, 39] and further support the recommendation of next‐generation ROS1‐TKIs as the preferred therapeutic option after crizotinib progression, especially in patients with ROS1 secondary mutations.

However, in real‐world settings, the use of next‐generation ROS1 inhibitors remains limited by their cost and accessibility. Consequently, other non‐next‐generation ROS1 inhibitors, such as entrectinib and lorlatinib, are frequently employed in postcrizotinib resistance settings because of their relatively greater accessibility. The efficacies of entrectinib and lorlatinib following crizotinib resistance have only been reported separately in single‐arm studies. Preliminary findings indicated limited clinical responses to entrectinib [11], whereas lorlatinib [12, 13] appeared to be more promising. However, direct comparisons of these two agents are lacking. Given that entrectinib and lorlatinib are frequently selected by clinicians in real‐world practice, we performed a comparative analysis to validate prior assumptions. Our results confirmed the limited efficacy of entrectinib, whereas lorlatinib demonstrated significantly superior performance, particularly in patients with CNS metastases. Taken together with earlier reports [11, 12, 13], these findings corroborate the recommendation of lorlatinib rather than entrectinib as the preferred therapy following crizotinib progression, particularly in cases of intracranial disease involvement.

Additionally, we explored the efficacy of PD‐1/PD‐L1‐based combination therapies in ROS1‐positive NSCLC patients. Although this regimen demonstrated numerically higher efficacy compared with IO alone in the IMMUNOTARGET study [35], our results revealed no significant advantage over conventional chemotherapy combined with anti‐VEGF therapy, indicating that chemotherapy remains an essential treatment option in later‐line settings. Moreover, further research is required to identify potential biomarkers predictive of immunotherapy efficacy in patients harboring driver oncogene mutations.

Additionally, we explored the efficacy of a PD‐1/PD‐L1‐based combination therapy in patients with ROS1‐positive NSCLC. Although this regimen demonstrated a numerically higher efficacy than IO alone in the IMMUNOTARGET study [35], our results revealed no significant advantage over conventional chemotherapy combined with anti‐VEGF therapy, indicating that chemotherapy remains an essential treatment option in later‐line settings. Further research is required to identify potential biomarkers predictive of IO efficacy in patients harboring driver oncogene mutations.

Our study has several limitations. First, because this was a retrospective analysis, selection and follow‐up biases were inevitable. Although we consecutively enrolled patients from January 2015 to January 2024 across the participating centers, many patients lacking detailed medical records or regular imaging follow‐up were excluded. Second, a central imaging assessment was not performed, and the evaluation intervals were not standardized for all patients. Additionally, regular brain magnetic resonance imaging (MRI) examinations were not mandated for patients without baseline CNS metastases, potentially leading to an evaluation bias. Third, the variability in the NGS and PD‐L1 testing platforms used across centers may have introduced confounding effects into our biomarker analyses. Fourth, intracranial efficacy was assessed using the RECIST criteria rather than specialized CNS‐focused criteria (e.g., mRECIST or RANO‐BM), possibly underestimating the intracranial tumor burden. Fifth, in the analysis of potential resistance mechanisms, paired baseline samples collected before crizotinib treatment were unavailable, and the diverse sample types collected during disease progression could introduce further confounding effects. Sixth, when assessing the efficacy of subsequent therapies, differences related to varying treatment lines were not accounted for and thus could represent additional confounding factors. Finally, all statistical analyses were limited by the sample size and absence of a predefined statistical hypothesis; therefore, these results should be interpreted cautiously.

## 4 Materials and Methods

### 4.1 Patient Selection

Our study was a single‐arm observational real‐world analysis. Patients diagnosed with locally advanced or metastatic ROS1‐positive NSCLC (AJCC eighth edition) who received crizotinib as the initial ROS1 inhibitor were enrolled from 17 hospitals across China between January 2015 and January 2024. Patients with symptomatic or active CNS metastases were eligible for inclusion. All enrolled patients were required to have comprehensive medical records, including detailed baseline radiological evaluations (brain MRI, bone scintigraphy [ECT], and computed tomography [CT] of the neck, thorax, and abdomen). During follow‐up, the enrolled patients underwent radiological assessments at least once every 6 months. A flowchart of the included patients is shown in Figure S1. Regular follow‐up brain MRI was not mandated for patients without baseline CNS metastases. Continuation of crizotinib treatment after localized or gradual disease progression was allowed at the discretion of the clinician. Patients with concomitant advanced malignancies were excluded.

### 4.2 Data Extraction

All data were extracted from the EMRs at each participating center. The demographic and clinical characteristics of the patients were systematically recorded. The specific ROS1 fusion partner was identified using NGS performed either at baseline or during disease progression. Additionally, PD‐L1 expression levels before initiating crizotinib treatment were documented, if available, in the EMRs. Results from biopsies and NGS analyses conducted at baseline or upon resistance development were also collected from the EMRs to explore potential efficacy biomarkers or resistance mechanisms. It should be noted that this study did not ensure consistent detection coverage or accuracy across the different NGS platforms. The initial radiological evaluation results were extracted from the EMRs; however, the imaging data were subsequently reviewed by the authors. The final radiological assessments reported in this study were based on the authors' evaluations rather than the original medical records. Survival outcomes were obtained from clinical records or telephone follow‐ups conducted by the investigators at each center. The data cutoff date was May 30, 2024. The last available follow‐up date was considered the data cutoff for patients lost to follow‐up before this date.

### 4.3 Definition and Assessment

Metastases occurring in symmetrical organs, such as the lungs, adrenal glands, bones, and nonregional lymph nodes, were considered to involve a single distant organ. Simultaneous brain RT was defined as RT administered within 3 months before or concurrently with crizotinib treatment. Radiological evaluation of tumor response was conducted based on the RECIST 1.1. Complete response (CR) and partial response (PR) comprised the ORR. PFS was defined as the interval between the initiation of crizotinib and the first documented radiological progression; local progression was defined as progressive disease (PD) involving ≤3 lesions confined to a single organ.

In our study, intracranial response was also assessed using RECIST 1.1, which was more practical and convenient, allowing selection of up to two intracranial target lesions (≥1 cm measured by MRI). The CNS‐ORR was defined as the achievement of CR or PR for the intracranial target lesions. The CNS tumor shrinkage rate was measured as the percentage reduction in the target lesion size at the best‐response assessment. CNS progressive disease was defined as follows: for patients with measurable CNS target lesions, progression was indicated by at least a 20% increase and an absolute increase of ≥5 mm in the sum of the largest diameters, or by the appearance of new CNS lesions; for patients without measurable CNS target lesions, progression was defined by significant enlargement (≥5 mm) of at least one nontarget CNS lesion or the development of new CNS lesions. CNS‐PFS was assessed exclusively in patients with CNS metastases and calculated from the initiation date of crizotinib to the date of documented CNS progression. It should be noted that some patients might experience extracranial progression while maintaining stable CNS disease. In such scenarios, overall PFS events were recorded as progression, whereas CNS‐PFS events were censored. Finally, OS was defined as the period from crizotinib treatment initiation to death from any cause. The primary study endpoints were PFS and CNS‐PFS rates for crizotinib.

### 4.4 Statistical Analysis

Given the observational nature of this study, no formal statistical hypotheses were formulated. Statistical analyses were conducted using the SPSS software (version 26.0; SPSS, Inc., Chicago, IL, USA). The patient distribution, baseline demographics, and clinical characteristics were summarized using descriptive statistics. The ORR for intracranial and extracranial lesions was calculated along with their corresponding 95% CIs based on the exact binomial distribution. Comparisons between groups were performed using Pearson's χ^2^ test for categorical variables and the *t*‐test for continuous variables. Survival curves were generated using the Kaplan–Meier method, and differences among groups were assessed using the log‐rank test. Cox proportional hazards regression analysis was used to calculate HRs and corresponding 95% CIs for selected covariates with the aim of identifying predictive factors associated with PFS. Statistical significance was defined as a two‐sided *p*‐value of < 0.05.

## Author Contributions

Concept and design: Zihua Zou and Gen Lin. Acquisition, analysis, or interpretation of data: Zihua Zou, Xiaobin Zheng, Panwen Tian, Zhe Liu, Jie Hu, Yong Fan, Yang Xia, Feng Ye, Tangfeng Lv, Li Li, Diansheng Zhong, Jin Zhou, Qian Chu, Meiqi Shi, Chengbo Han, Baoshan Cao, Dingzhi Huang, Hui Guo, and Gen Lin. Drafting of the manuscript: Zihua Zou and Gen Lin. Critical review of the manuscript for important intellectual content: Zihua Zou, Xiaobin Zheng, Panwen Tian, Zhe Liu, Jie Hu, Yong Fan, Yang Xia, Feng Ye, Tangfeng Lv, Li Li, Diansheng Zhong, Jin Zhou, Qian Chu, Meiqi Shi, Chengbo Han, Baoshan Cao, Dingzhi Huang, Hui Guo, and Gen Lin. Statistical analysis: Zihua Zou and Xiaobin Zheng. All authors have read and approved the final manuscript.

## Funding

This study was funded by the National Natural Science Foundation of China (No. 82372954) and the Beijing Chest Hospital Talent Recruitment Scientific Research Start‐up Project (No. 1‐745)

## Ethics Statement

This study was conducted in accordance with the Declaration of Helsinki (as revised in 2013), approved by the Ethics Committee of Fujian Cancer Hospital (K2023‐450‐01), and informed consent was obtained from all participants.

## Conflicts of Interest

All authors have no conflicts of interest to declare.

## Supporting information




**Figure S1**: Included patients flow chart. **Figure S2A**: Intracranial metastases and associated cerebral edema nearly resolved following treatment with crizotinib. **Figure S2B**: The patient's intracranial lesions significantly decreased in size and number following crizotinib treatment. **Figure S2C**: CNS lesions nearly resolved following crizotinib treatment.

## Data Availability

Individual data will be made available following publication by reasonable request to the corresponding author.
