# Supplementary material for: Intracranial Efficacy of Crizotinib and Postprogression Therapeutic Strategies in Advanced c‐ros Oncogene 1 (ROS1)‐Positive Non–Small Cell Lung Cancer (NSCLC), a Multicenter Real‐World Study
Source: MedComm (2020). 2026 Jul 13;7(7):e70854. doi: 10.1002/mco2.70854 (PMC13358559; doi:10.1002/mco2.70854)
Supplement: Supplementary file 1 — Figure S1: Included patients flow chart. Figure S2A: Intracranial metastases and associated cerebral edema nearly resolved following treatment with crizotinib. Figure S2B: The patient's intracranial lesions significantly decreased in size and number following crizotinib treatment. Figure S2C: CNS lesions nearly resolved following crizotinib treatment. [file MCO2-7-e70854-s001.docx]

**Intracranial efficacy of crizotinib and post-progression therapeutic strategies in advanced c-ros oncogene 1 (ROS1) -positive non-small cell lung cancer (NSCLC) a multicenter real-world study**

**Authors**

**Zihua Zou^1^**#**, Xiaobin Zheng^2^**#**, Panwen Tian^3^**#**, Zhe Liu^2^**#**, Jie Hu^4^**#**, Yong Fang^5^, Yang Xia^6^, Feng Ye^7^, Tangfeng Lv^8^, Li Li^9^, Diansheng Zhong^10^, Jin Zhou^11^, Qian Chu^12^, Meiqi Shi^13^, Chengbo Han^14^, Baoshan Cao^15^, Dingzhi Huang^16^, Hui Guo^17^, Gen Lin^2^***

1.Department of Thoracic Oncology, Clinical Oncology School of Fujian Medical University, Fujian Cancer Hospital

2.Department of Medical Oncology, Beijing Tuberculosis and Thoracic Tumor Research Institute, Beijing Chest Hospital, Capital Medical University

3.Department of Pulmonary and Critical Care Medicine, Lung Cancer Center, West China Hospital, Sichuan University, Precision Medicine Key Laboratory of Sichuan Province

4.Zhongshan Hospital，Fudan University, Shanghai Geriatric Center

5.Department of Medical Oncology, Sir Run Run Shaw Hospital, Zhenjiang University School of Medicine

6.Department of Respiratory and Critical Care Medicine, Second Affiliated Hospital of Zhejiang University School of Medicine

7.Department of Medical Oncology, Xiamen Key Laboratory of Antitumor Drug Transformation Research, the First Affiliated Hospital of Xiamen University, School of Medicine

8.Department of Respiratory Medicine, Affiliated jinling Hospital,Medical School of Nanjing University

9.Department of Respiratory Disease, Daping Hospital, Army Medical University

10.Department of Medical Oncology, Tianjin Medical University General Hospital

11.Department of Medical Oncology, Sichuan Clinical Research Center for Cancer, Sichuan Cancer Hospital & Institute, Sichuan Cancer Center, Affiliated Cancer Hospital of University of Electronic Science and Technology of China

12.Department of Oncology, Tongji Hospital, Tongji Medical College, Huazhong University of Science and Technology

13.Department of Medical Oncology, Jiangsu Cancer Hospital and Jiangsu Institute of Cancer Research and Affiliated Cancer Hospital of Nanjing Medical University

14.Department of Oncology, Shengjing Hospital of China Medical University

15.Cancer center, Peking University Third Hospital/ Department of medical oncology and radiation sickness, Peking University Third Hospital

16.Department of Thoracic Oncology, Tianjin Medical University Cancer Institute & Hospital

17.Department of Medical Oncology, the Second Affiliated Hospital of Xi'an Jiaotong University

Zihua Zou, Xiaobin Zheng, Panwen Tian, Zhe Liu, Jie Hu contributed equally to this research

Corresponding author: Gen Lin e-mai: lingen@bjxkyy.cn


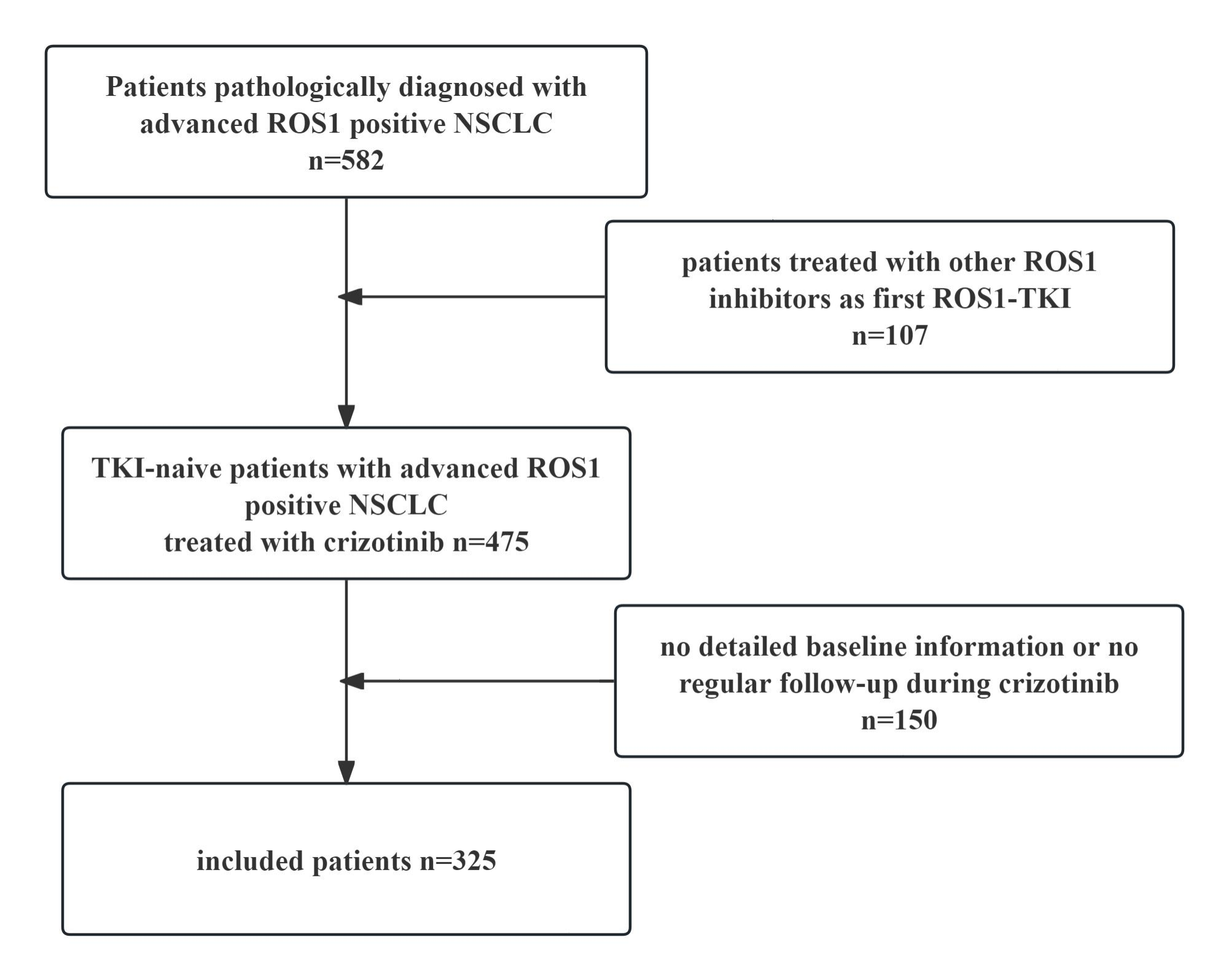


**Figure S1 Included patients flow chart**


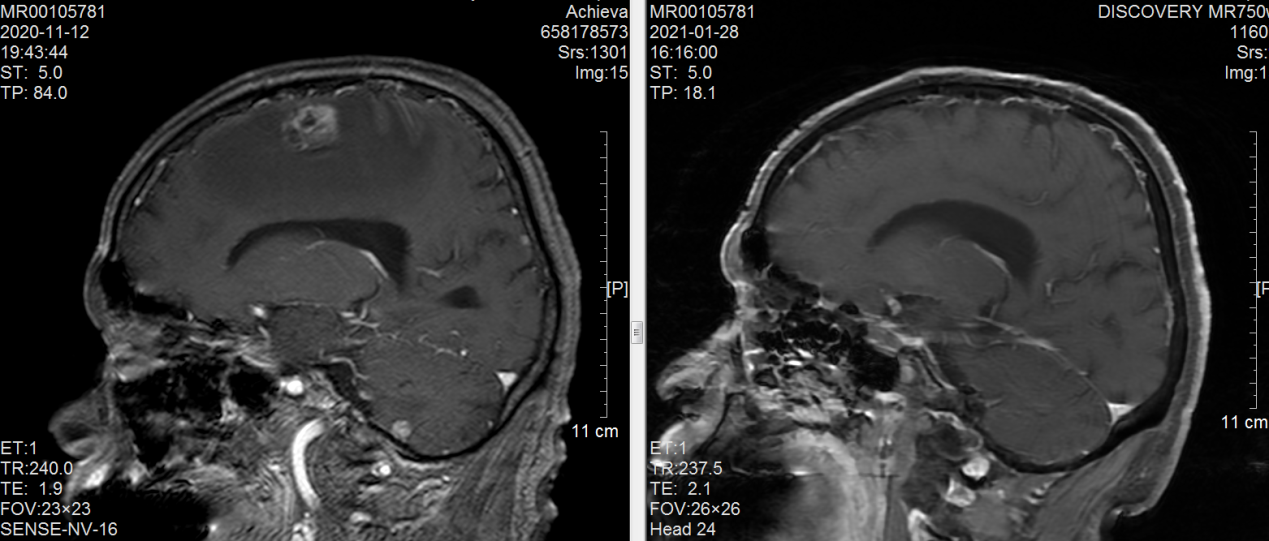


**Figure S2A Intracranial metastases and associated cerebral edema nearly**

**resolved following treatment with crizotinib**


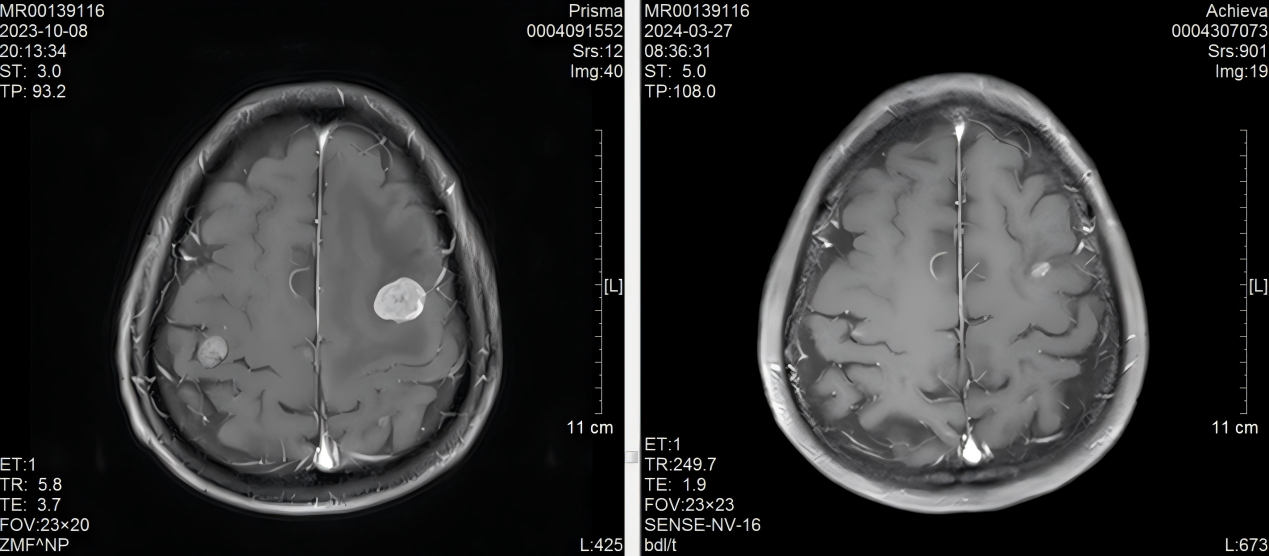


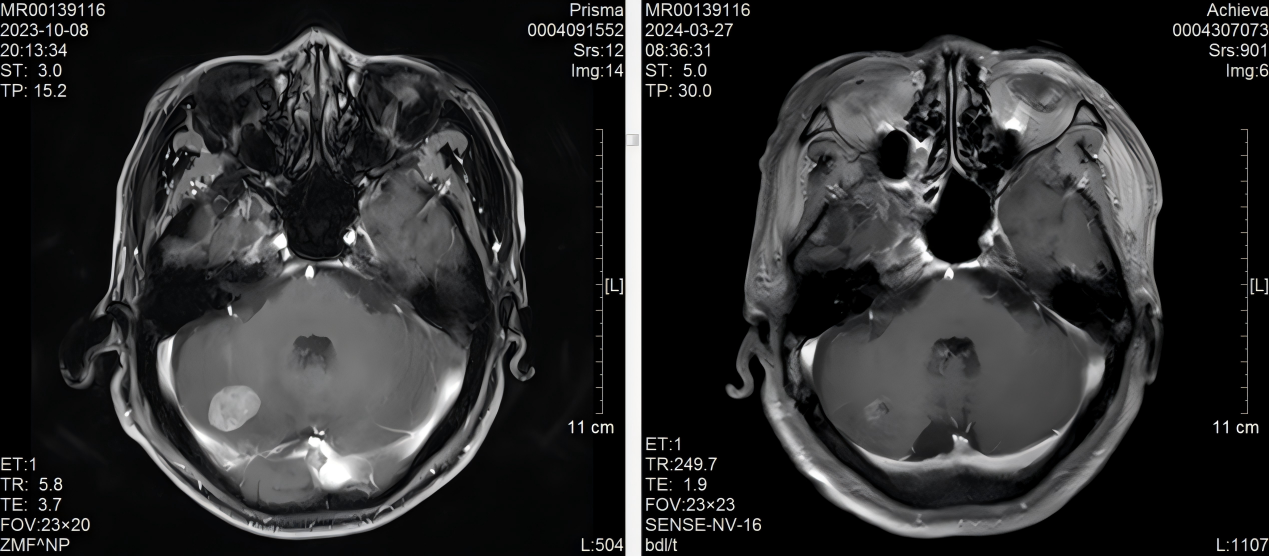


**Figure S2B The patient's intracranial lesions significantly decreased in size**

**and number following crizotinib treatment**


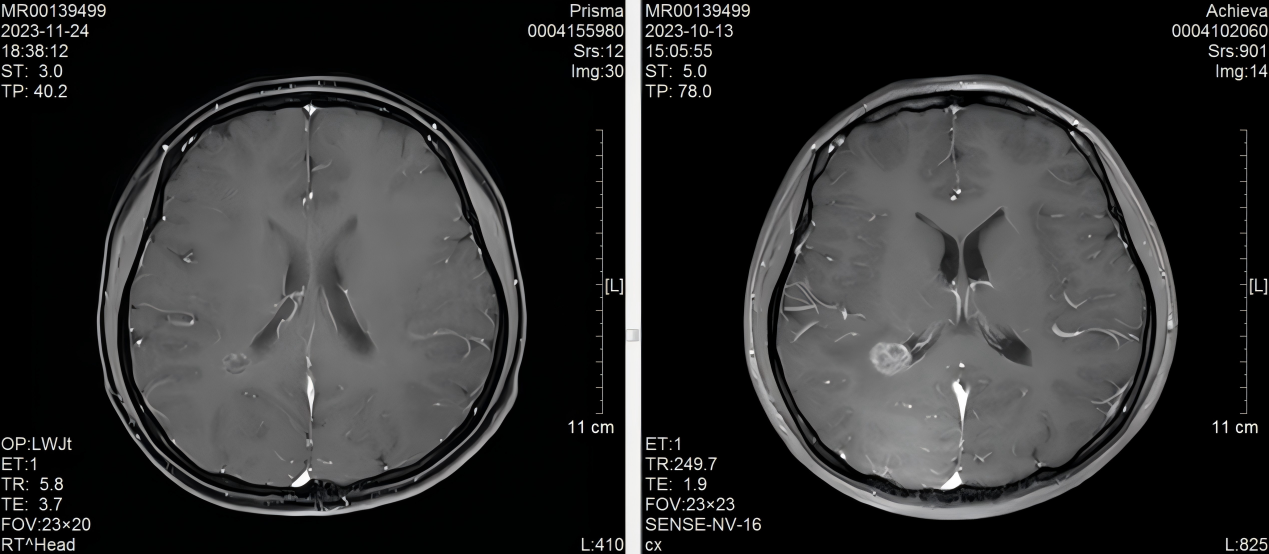


**Figure S2C CNS lesions nearly resolved following crizotinib treatment**
